# The Role of Information Boxes in Search Engine Results for Symptom Searches: Analysis of Archival Data

**DOI:** 10.2196/37286

**Published:** 2022-09-14

**Authors:** Lorien C Abroms, Elad Yom-Tov

**Affiliations:** 1 Milken Institute School of Public Health George Washington University Washington, DC United States; 2 Microsoft Research Israel Hertzliya Israel

**Keywords:** health misinformation, search engine, internet search, information boxes, knowledge graph boxes, misinformation, health information, Microsoft, internet, data, symptoms, results, users, medical, Bing, USA, linear, logistic, regression, web, ads, behavior

## Abstract

**Background:**

Search engines provide health information boxes as part of search results to address information gaps and misinformation for commonly searched symptoms. Few prior studies have sought to understand how individuals who are seeking information about health symptoms navigate different types of page elements on search engine results pages, including health information boxes.

**Objective:**

Using real-world search engine data, this study sought to investigate how users searching for common health-related symptoms with Bing interacted with health information boxes (info boxes) and other page elements.

**Methods:**

A sample of searches (N=28,552 unique searches) was compiled for the 17 most common medical symptoms queried on Microsoft Bing by users in the United States between September and November 2019. The association between the page elements that users saw, their characteristics, and the time spent on elements or clicks was investigated using linear and logistic regression.

**Results:**

The number of searches ranged by symptom type from 55 searches for cramps to 7459 searches for anxiety. Users searching for common health-related symptoms saw pages with standard web results (n=24,034, 84%), itemized web results (n=23,354, 82%), ads (n=13,171, 46%), and info boxes (n=18,215, 64%). Users spent on average 22 (SD 26) seconds on the search engine results page. Users who saw all page elements spent 25% (7.1 s) of their time on the info box, 23% (6.1 s) on standard web results, 20% (5.7 s) on ads, and 10% (10 s) on itemized web results, with significantly more time on the info box compared to other elements and the least amount of time on itemized web results. Info box characteristics such as reading ease and appearance of related conditions were associated with longer time on the info box. Although none of the info box characteristics were associated with clicks on standard web results, info box characteristics such as reading ease and related searches were negatively correlated with clicks on ads.

**Conclusions:**

Info boxes were attended most by users compared with other page elements, and their characteristics may influence future web searching. Future studies are needed that further explore the utility of info boxes and their influence on real-world health-seeking behaviors.

## Introduction

A general-purpose internet search engine is the first stop for most people who experience a health symptom and are seeking information about it [1-3]. The search engine results page (SERP), provided by search engines, generally includes a variety of page elements. These include the standard search results with a URL and a summary or snippet. Additionally, the search results page may include other page elements such as videos, advertisements, recent news stories, and in the case of health-related searches, a health information box [4,5].

**Figure 1:**
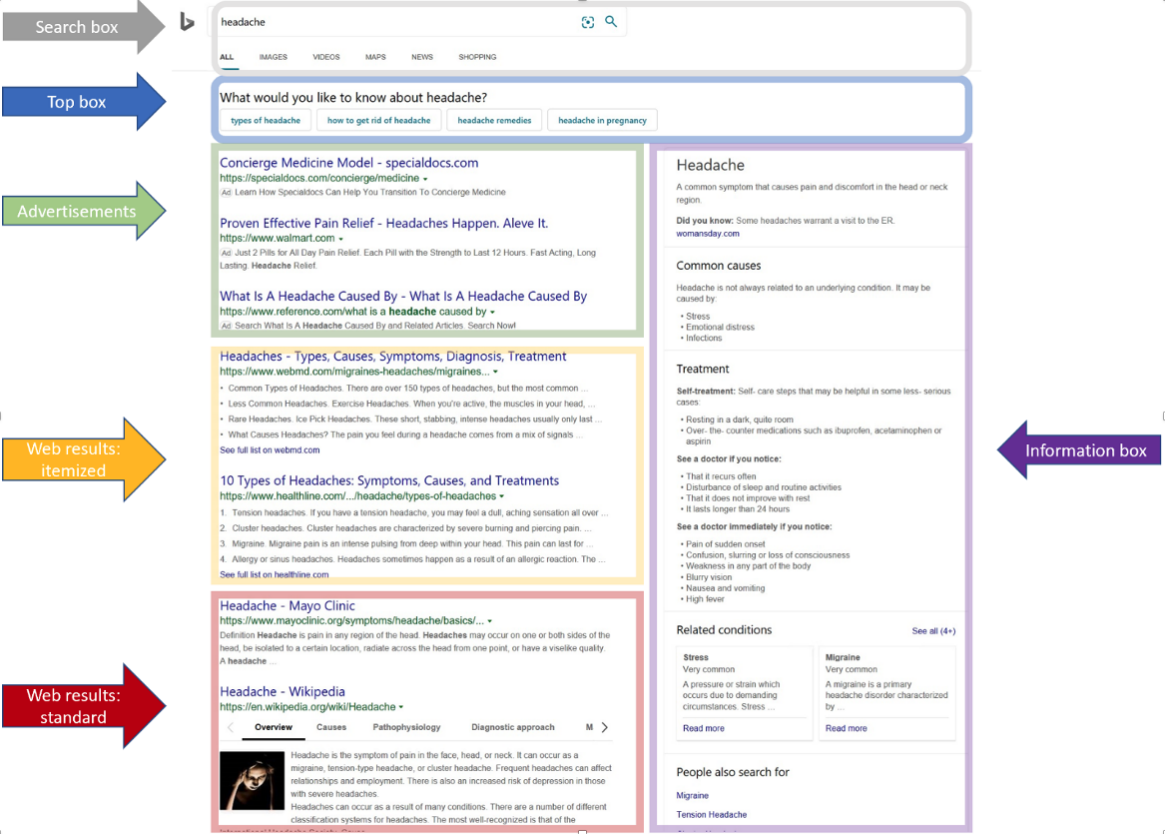
Typical search engine results page for “headache” with multiple page elements displayed, including info box, ads, itemized web results, and standard web results.

Health information boxes (info boxes), also known as health knowledge graph boxes, information cards, or task panes, were created at major search engines about 10 years ago—at Bing in 2010 and at Google in 2012 [6]. They were developed to address health information gaps and misinformation for commonly searched symptoms that might arise from search results alone [4]. Info boxes are typically presented in the right-hand side of a SERP and are available in addition to what is available from the standard search results (as seen in Figure 1). Info boxes could balance the information presented in search results that might otherwise lead a user to, for example, overworry about a symptom (eg, headache) based on standard search results alone [7]. The information in info boxes is provided by the search engine from sources they deem trustworthy (eg, Mayo Clinic and Wikipedia) and may have additional reviews from an internal health team [7].

Few prior studies have sought to understand how individuals who are seeking health information navigate SERPs and their various page elements such as standard search results, ads, or videos (exceptions include [4,5,8]). However, understanding how users interact with page elements is a fundamental question in information retrieval, with implications for understanding search quality and interface design. In the case of symptom search, these have implications for health knowledge acquisition, methods of addressing information gaps and misinformation, as well as future health-seeking behaviors, potentially. Past studies have found that a search engine’s sorting and ranking criteria can directly influence engagement, user effort, as well as health beliefs and attitudes [2,8]. The salience on search results page may also affect the decision to present to health services [2].

Despite their long-standing existence and ubiquity, only one study could be identified that had examined the role of info boxes. A study by Ludolph et al [4] found that experimentally developed and manipulated info boxes (termed knowledge graph boxes in the study), which were shown as part of a web-based survey, could positively affect a participants’ vaccination-related knowledge and attitudes. No study that we are aware of has previously sought to understand the effects of info boxes using real-world data or in the context of health symptom searches.

This study sought to investigate how users searching for common health-related symptoms with Bing interacted with info boxes and other page elements using real-world data collected from anonymized Bing users. The research question under investigation was whether curated content on health symptoms as presented in the info boxes affected health-seeking behavior by Bing users, and to what extent info boxes and other page elements attended to and used in the SERPs were compared with other page elements.

## Methods

We compiled a list of the 19 most common medical symptoms queried on Bing by users in the United States between September and November 2019 from a longer list of 195 symptoms originally compiled from Wikipedia in a prior study [9]. The list was refined to remove 2 items identified by our team as not being symptoms (ie, childbirth and weight loss). The remaining list was comprised of 17 symptoms as follows: anxiety, back pain, bleeding, constipation, cough, cramp, depression, diarrhea, fever, headache, itch, pain, paralysis, rash, wound, swelling, and tremor.

To obtain the sample of searches for these symptoms, deidentified data on symptom searches made on Bing in the United States during September 2019 were extracted. We also extracted information about the interaction of the users with the search results page (that is explained in more detail in the following paragraph). Our sample was comprised of a total of 33,872 searches for the 17 symptoms, encompassing 28,552 unique users. We limited our sample to the first search of users in order to have a sample where each search was independent. Thus, 28,552 searches were included for analysis and comprise the final sample. The distribution of symptoms among searches is shown in Table 1.

Search-related information on each user included the following: page elements shown to the user on the SERP, clicks on any of the displayed elements on the SERP, and the time that the mouse pointer spent on each of the elements, previously shown to be a marker for attention [10].

**Table 1:** The number of searches per symptom in the analyzed data set.

Symptoms	Number of searches
Anxiety	6686
Back pain	1576
Bleeding	136
Constipation	2558
Cough	674
Cramp	100
Depression	5485
Diarrhea	5784
Fever	899
Headache	682
Itch	395
Pain	1371
Paralysis	295
Rash	927
Swelling	236
Tremor	345
Wound	403
Total	28,552

A typical SERP had the following page elements: advertisements, which are created by external parties who pay whenever they are clicked; a health information box or “info box,” which contains Bing-curated health information; and two areas where algorithmic search answers are shown—one containing standard web results described by several sentences of text or snippets and the other containing an itemized web result with a summary of information. The standard web results are ranked based on the result predicted to be most relevant (rank=1 for highest position on the page) to least relevant (rank=8), though not all results may be displayed on the first page.

In addition, a typical SERP may have a top box, which helps disambiguate the user’s intent by offering more focused search options or providing information such as dictionary definitions. Other elements that are sometimes displayed on the page include video results and news. However, as data for these elements (ie, top box, video elements, and news) were not readily available for extraction, these page elements were excluded from our analysis. Thus, we restricted our analysis to the following page elements: ads, info boxes, standard web results, and itemized web results. It is noteworthy that not all page elements are shown to each user for a given search, and results displayed may depend on factors such as the size of their browser window. Figure 1 shows a sample symptom SERP, displaying its different page elements.

We manually coded the characteristics of the info boxes associated with these commonly searched symptoms. In order to display the info boxes for coding, the symptom name was typed into the Bing search engine with a fresh private (ie, incognito) window using the Microsoft Explorer web browser. Info boxes were coded for reading ease (using the Flesch Reading Ease score, with higher scores on a scale of 0-100 indicating greater ease of reading). They were also coded for whether the info box shows related searches (eg, common causes and treatment) or provides information on related conditions.

In addition, we manually coded the characteristics of ads and the standard search results. For this, the 20 most commonly displayed ads and search results associated with each of the symptoms in September 2019 were identified and manually coded by a single coder. Ads and search results were scored for reading level (using the Flesch Reading Ease score) and coded for the type of information offered (eg, informational or product advertisement). A random subsample of 50 ads and 50 web results were independently coded by a second coder for type of information—the most subjective of the codes.

User engagement with the elements on the page was measured as the time spent on each of the page elements (eg, ads, info box, itemized web results, and standard web results) and whether itemized web results and standard web results were clicked. Times were measured by monitoring whether the mouse pointer of the user was hovering over an element [10]. The total time on a page included the entire time that the user spent with a search result, including any returns to it following a visit to one of the search results.

Descriptive statistics were tabulated for engagement metrics (eg, seconds on page elements and clicks on page elements). Linear regression was used to analyze the correlation between the time spent on different elements of the page, as a function of the elements shown on the page. Logistic regression was used to analyze the association between page characteristics, info box characteristics, the characteristics of standard web results, and (separately) those of ads on clicks on standard web results or ads. This analysis was conducted at the level of a standard web result or ad. We did not analyze clicks on itemized web results, as clicks on them were the least common. We did not analyze clicks on info boxes, as many of them did not have links, and therefore, clicks were rare.

## Results

For the subsample analyzed, kappa statistics for the agreement between the coders was generally good for the type of information in ads (κ=0.60) and standard web results (κ=0.44). Among the 28,552 symptom searches of unique individuals analyzed, the number of searches ranged by symptom type from 55 searches for cramps to 7459 searches for anxiety. Users searching for symptoms encountered SERPs with multiple page elements, including standard web results (n=24,034, 84%), itemized web results (n=23,354, 82%), ads (n=13,171, 46%), and info boxes (n=18,215, 64%; Table 2).

When all the 4 elements of the page (ie, info box, ads, itemized web results, and standard web results) were shown to users, 41% (2039) of them went on to click on some elements in the SERP, with the remainder not clicking on anything. Users clicked on standard web results most often (ie, n=4612, 19%), and they clicked on ads 12% (n=1633) of the time. They clicked on itemized web results least often (n=1798, 8%).

On average, users spent 22 (SD=26) seconds on the SERP once the results were shown to them, with 24% (n=1182) spending 30 seconds or more on it. As Table 2 demonstrates, users who saw all page elements spent 25% (7.1 s) of their time on the info box, 23% (6.1 s) on standard web results, 20% (5.7 s) on ads, and 10% (10 s) on itemized web results, with significantly more time on info boxes compared to other elements and the least amount of time on itemized web results (sign test; all pairwise comparisons are statistically significant; *P*<.001).

Based on manual coding, the info boxes were found to have the following characteristics: the average Flesch Reading Ease score of info boxes was 46 (SD 17; range 6-69); common causes and treatment of the symptom were shown in 76% (n=13) of the info boxes; the info boxes contained a list of related conditions in 71% (n=12) of the cases, and related searches were shown for all but one symptom (diarrhea); the most common data source (as stated in the info boxes) for the information in the info boxes was Focus Medica (n=14), with the remainder citing Wikipedia as their data source (n=3).

The time spent on the info box was modeled using linear regression, as a function of the coded characteristics of the info box. The model fit was *R*^2^=0.016 (*P*<.001; n=17,255), meaning that the characteristics of the info box (eg, reading ease, showing related conditions, and showing related searches) is associated with time spent on the info box, but the characteristics explain only a small amount of the variance in time. That said, the appearance of related conditions and ease of reading were significantly associated with longer time, whereas related searches were correlated with shorter time.

Table 3 shows a model of the time spent on an element (in seconds), as a function of whether the other elements of the page were visible. As the model shows, there is a weak correlation between the time spent and the visibility of other elements. Longer time spent on the info box is most strongly associated with the display of itemized web results and ads. Longer time spent on itemized web results is most strongly associated with the display of ads and info box and negatively correlated with the display of itemized web results. Longer time spent on standard web results is associated most strongly with the display of itemized web results and ads.

Table 4 shows logistic regression models for predicting clicks on individual standard web results and ads, taking into account the characteristics of the info box, the characteristics of the page, and those of the standard web result, or the ad. Characteristics of the info boxes include the following: whether they were shown; and if so, whether related conditions were shown; whether related searches were shown; and the reading ease of these boxes. Characteristics of the page include the time the mouse pointer hovers over ads, info boxes, and the two types of web links, as well as the number of elements in each type. The attributes of the web results and ads include whether they were informational or advertisements, their reading ease, the rank at which they were shown on the page (1 being the highest rank), and the time that the mouse pointer hovered over the link.

As can be seen in Table 4, when an info box was shown, an info box showing related conditions was associated with higher likelihood of clicks on ads. Related searches and reading ease were negatively correlated with clicks on ads. None of the parameters of info boxes were associated with clicks on standard web results.

Being shown more ads was associated with more clicks on ads, but it was unrelated to clicking on standard web results, while more standard web results shown were associated with fewer clicks on those results or ads. Standard web results with informational content were less likely to be clicked.

**Table 2:** Statistics of page elements during symptom searches (N=28,552).

Page elements	Visible to the user, n (%)	Clicks on visible elements, n (%)	Time spent (when all elements are shown), seconds (%)
Ads	13,171 (46)	1633 (12)	5.7 (20)
Info box	18,215 (64)	N/A^a^	6.1 (25)
Itemized web results	23,354 (82)	1798 (8)	2.7 (10)
Standard web results	24,034 (84)	4612 (19)	7.1 (23)

^a^N/A: not applicable. Info boxes are not usually clicked, and therefore, this number is not given.

**Table 3:** Model for predicting time spent on different elements of the page, as a function of the elements shown on the page. Numbers shown are model slopes.

Page elements	Model *R*^2^	Elements shown			
		Ads	Info boxes	Itemized web results	Standard web results
Ads	0.001	—^a^	–0.020^b^	–0.700	–0.012^b^
Info boxes	0.037	1.413	—	1.654	2.189
Itemized web results	0.009	0.495	0.734	—	–0.343
Standard web results	0.014	1.143	0.961	2.121	—

^a^Not applicable.

^b^Slopes that are not statistically significant (at *P*<.05, with Bonferroni correction).

**Table 4:** Logistic regression models of clicks on individual standard web results and on individual ads in cases where the information box (info box) was shown.

Characteristics		Standard web results (n=23,776), OR^a^ (95% CI)	Ads (n=16,667), OR (95% CI)
**Info box**			
	Info box shows related conditions	1.281 (1.053-1.557)	1.331^b^ (1.107-1.599)
	Info box shows related searches	0.985 (0.839-1.156)	0.634^b^ (0.559-0.718)
	Info box’s reading ease	0.997 (0.994-1.000)	0.996^b^ (0.994-0.998)
**Page**			
	Ad’s rank	1.007 (0.983-1.031)	0.988 (0.973-1.005)
	Number of ads shown	1.015 (0.998-1.032)	1.058^b^ (1.031-1.086)
	Number of itemized web results shown	0.989 (0.956-1.023)	0.928 (0.874-0.984)
	Number of standard web results shown	0.901^b^ (0.884-0.918)	0.881^b^ (0.862-0.901)
	Time spent on ads	1.009^b^ (1.005-1.013)	0.999 (0.994-1.003)
	Time spent on info boxes	0.995 (0.989-1.000)	0.996 (0.988-1.003)
	Time spent on itemized web results	0.997 (0.990-1.004)	1.007 (0.999-1.016)
	Time spent on standard web results	0.992 (0.986-0.997)	1.004 (0.999-1.009)
**Standard web result or ad**			
	Type of information (informational)	0.789^b^ (0.693-0.899)	0.905 (0.785-1.044)
	Reading ease of elements	1.000 (0.999-1.002)	1.002 (1.000-1.004)
	Time spent on standard web results or ads	1.023 (1.005-1.041)	1.005 (0.999-1.009)

^a^OR: odds ratio.

^b^Ratios are statistically significant (at *P*<.05, with Bonferroni correction).

## Discussion

### Principal Findings

For people experiencing health symptoms, search engines have become a dominant way of initially making sense of that experience [1,3]. As such, understanding how individuals who are seeking information about health symptoms navigate different types of page elements, including info boxes, on SERPs is paramount.

This study of 28,552 unique Bing users searching for 17 common symptoms found that users searched most often for information on anxiety and least often for information on cramps. In doing those searches, users spent on average 22 seconds observing the SERP and encountered SERPs with a complex mix of ads, standard web results, itemized web results, and info boxes. Standard web results and itemized web results were most common in SERPs, and ads and info boxes were present fairly less often, about half of the time. The variation observed in what users saw was likely because of their specific search, the device they used to browse them (eg, screen size differences, with smaller screens displaying fewer content elements), and user behavior, in cases where the user did not scroll down to the location of that element.

When all page elements of the SERP were visible (ie, info box, ads, standard web results, and itemized web results), users spent the most time observing info boxes. This represents the first real-world evidence that info boxes are serving the purpose that they were designed to do, namely, presenting health information in a more user-friendly format compared to standard web results. Users may prefer info boxes over other types of SERP elements because they simplify the information and manage information overload.

Furthermore, info box characteristics were found to be associated with a decreased likelihood of clicking on ads, but they had no effect on standard web results. This implies that a well-designed info box—one that is higher on reading ease and shows related searches—may reduce the likelihood that those searching for health symptom information will be steered to commercial websites. As such, designers of info boxes may wish to carefully consider their design elements and ensure that the reading level is as low as possible. Furthermore, given their importance, search engine companies may wish to pretest their content with users or test out variations in order to optimize them.

The strength of this study is that it provides the first real-world data on symptom searches on search engines, and how users interact with info boxes. It includes real-world stimuli and data from real users searching on Bing. As this study is the first of its type, future studies are needed to confirm these findings as well as take them further by examining the real-world implications of SERPs for symptom searches. For example, studies could examine how info boxes affect future decision-making about whether to seek out medical care or pursue various treatment options.

Weaknesses of this study include the following: although we were able to examine multiple page elements from SERPs, we were not able to access the types of page elements presented to users; For example, we did not have access to data on top boxes that simplify search or in videos shown to users; future studies should strive to include these other data types. Additionally, the list of 17 symptoms investigated was generated from a longer list of 195 symptoms compiled by Wikipedia, which may be less reliable than other types of data on symptoms, such as population-level survey data.

### Conclusions

SERPs for symptom searches often include info boxes that are attended to by users. Info box characteristics may influence future web searching. Future studies are needed to further explore the utility of info boxes, how to optimize them, and their influence on real-world treatment-seeking behaviors.

## Abbreviations

SERP: search engine results page
